# Supplementary material for: Structure of HIV-1 RRE stem-loop II identifies two conformational states of the high-affinity Rev binding site
Source: Nat Commun. 2024 May 17;15:4198. doi: 10.1038/s41467-024-48162-y (PMC11101469; doi:10.1038/s41467-024-48162-y)
Supplement: Supplementary file 1 — Supplementary information [file 41467_2024_48162_MOESM1_ESM.docx]

**Supplementary Information for**

**Structure of HIV-1 RRE stem-loop II identifies two conformational states of the high-affinity Rev binding site**

Jerricho Tipo^1,2^, Keerthi Gottipati^2^, Michael Slaton^2^, Giovanni Gonzalez-Gutierrez^2^, Kyung H. Choi^1,2,3,*^

^1^Department of Pharmacology and Toxicology, The University of Texas Medical Branch, Galveston, TX 77555, USA

^2^Department of Molecular and Cellular Biochemistry, Indiana University, Bloomington, IN 47405, USA

^3^Department of Biochemistry and Molecular Biology, Sealy Center for Structural Biology, The University of Texas Medical Branch, Galveston, TX 77555, USA

^*^To whom correspondence should be addressed. Tel:812-855-1159; Email: [kaychoi@iu.edu](mailto:kychoi@utmb.edu)

Table S1-2

Figures S1-4

**Table S1. Superposition between the two RRE SLII conformers**

|  | **Full length SLII** | **Stem IIA** | **Stem IIB** | **Stem IIC** | **3-way junction** |
| --- | --- | --- | --- | --- | --- |
| No. of paired nt | 67 | 14 | 23 | 21 | 9 |
| RMS Deviation (Å) | 6.6 | 0.9 | 2.6 | 1.4 | 10.0 |

**Table S2. Crystal contacts of HIV RRE SLII-tRNA structure**

| **Chain A** | | **Chain B** | | Interactions |
| --- | --- | --- | --- | --- |
| Nucleotide | Atom | Nucleotide | Atom |  |
| A51 | N7 | G69 | N2 | H-bond* |
|  | N6 | G69 | O2’ | H-bond |
|  |  | G70 | O4 | H-bond |
| G52 | N3 | N68 | N1 | H-bond |
|  | O4 |  | N6 | H-bond |
| G53 | O6 | C67 | N4 | H-bond |
|  | N1 |  | N3 | H-bond |
|  | N2 |  | O2 | H-bond |
| A61 | N6 | A66 | OP2 | H-bond |
| U65 | O2 | G31 | O2’ | H-bond |
| A66 | O4’ | G30 | O2’ | H-bond |
| C67 | N4 | G53 | O6 | H-bond |
|  | N3 |  | N1 | H-bond |
|  | O2 |  | N2 | H-bond |
| A68 | N6 | U52 | O4 | H-bond |
|  | N1 |  | N3 | H-bond |
| G69 | N2 | A51 | N3 | H-bond |
|  |  |  | O2’ | H-bond |
| G70 | N3 | A51 | O2’ | H-bond |
| **Chain A** | | **Chain B’** ** | |  |
| G19 | G base | U20 | U base | Base stacking |
|  | O2’ |  | OP2 | H-bond |
|  | O3’ | G19 | O2’ | H-bond |
| U20 | U base | G19 | G base | Base stacking |
| G22 | O2’ | A61 | N6, N1 | H-bond |
| A23 | O2’ | A61 | N7 | H-bond |
| G31 | O2 | C86 | O2’ | H-bond |
| C76 | O3’ | G123 | O2’ | H-bond |
|  | O2’ |  | O2’ | H-bond |
| C86 | O2’ | G110 | O2’ | H-bond |
| G100 | O2’ | A77 | O2’ | H-bond |
| C101 | O2’ | A134 | O2’ | H-bond |
|  |  |  | O3’ | H-bond |
|  | O2 |  | O2’ | H-bond |
| C114 | C base | A81 | A base | Base stacking |
| U122 | OP1 | G1 | O2’ | H-bond |
| A134 | O2’ | G27 | N2 | H-bond |
|  |  |  | O2 | H-bond |
| **Chain A** | | **Chain A’** | |  |
| C13 | OP1 | G38 | N1 | H-bond |
| U79 | O4’ | C2 | O2’ | H-bond |
| **Chain B** | | **Chain B'** | |  |
| U20 | N3 | A81 | N1 | H-bond |
| A81 | N6 | C114 | O2’ | H-bond |

* H-bond distances < 3.5 Å are listed.

** Symmetry related molecules were denoted with ‘ symbol.


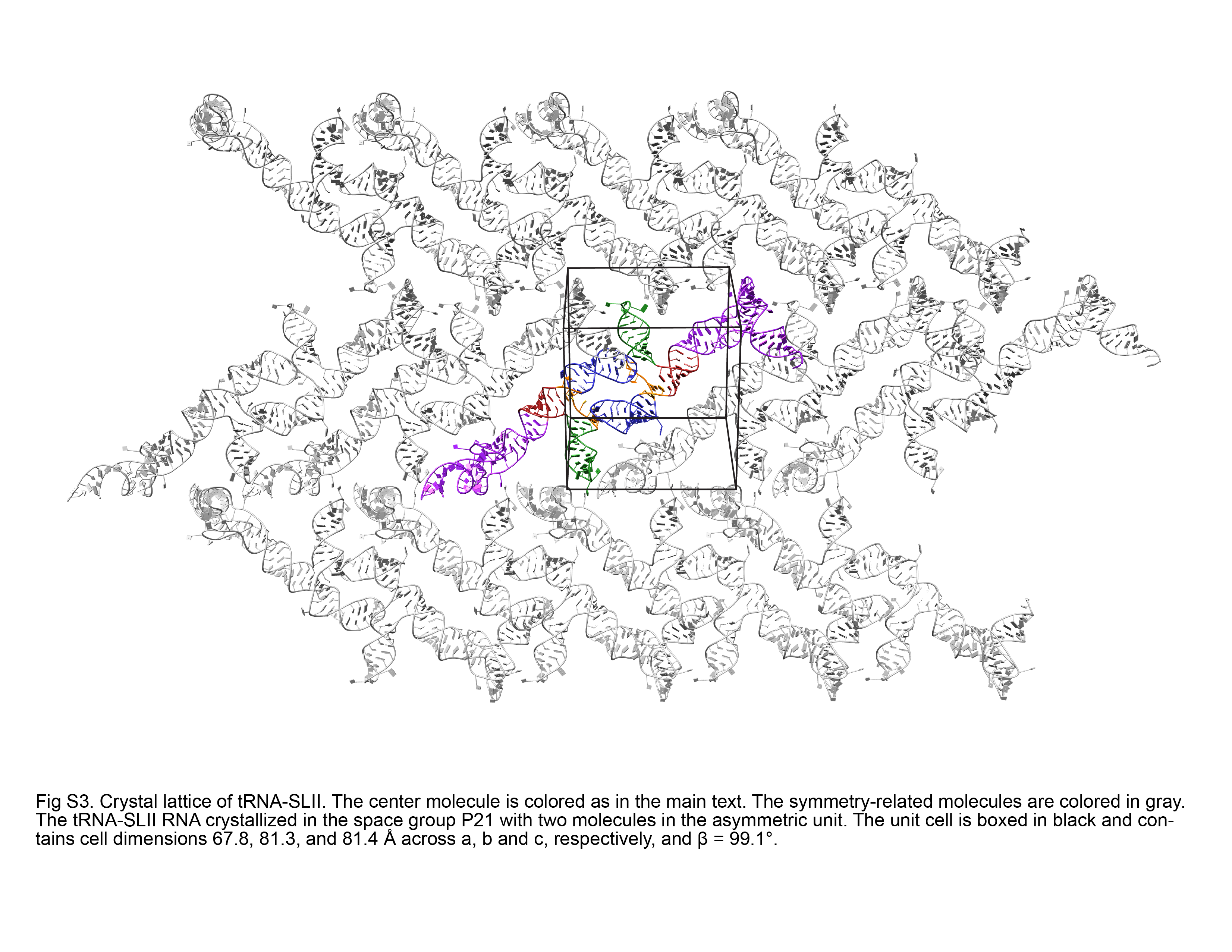


**Fig S1**. **Crystal lattice of tRNA-SLII**. The tRNA-SLII RNA crystallized in the space group P21 with two molecules in the asymmetric unit. The center molecule is colored as in Fig 1 and the symmetry-related molecules are colored in gray. The unit cell is boxed in black.

**
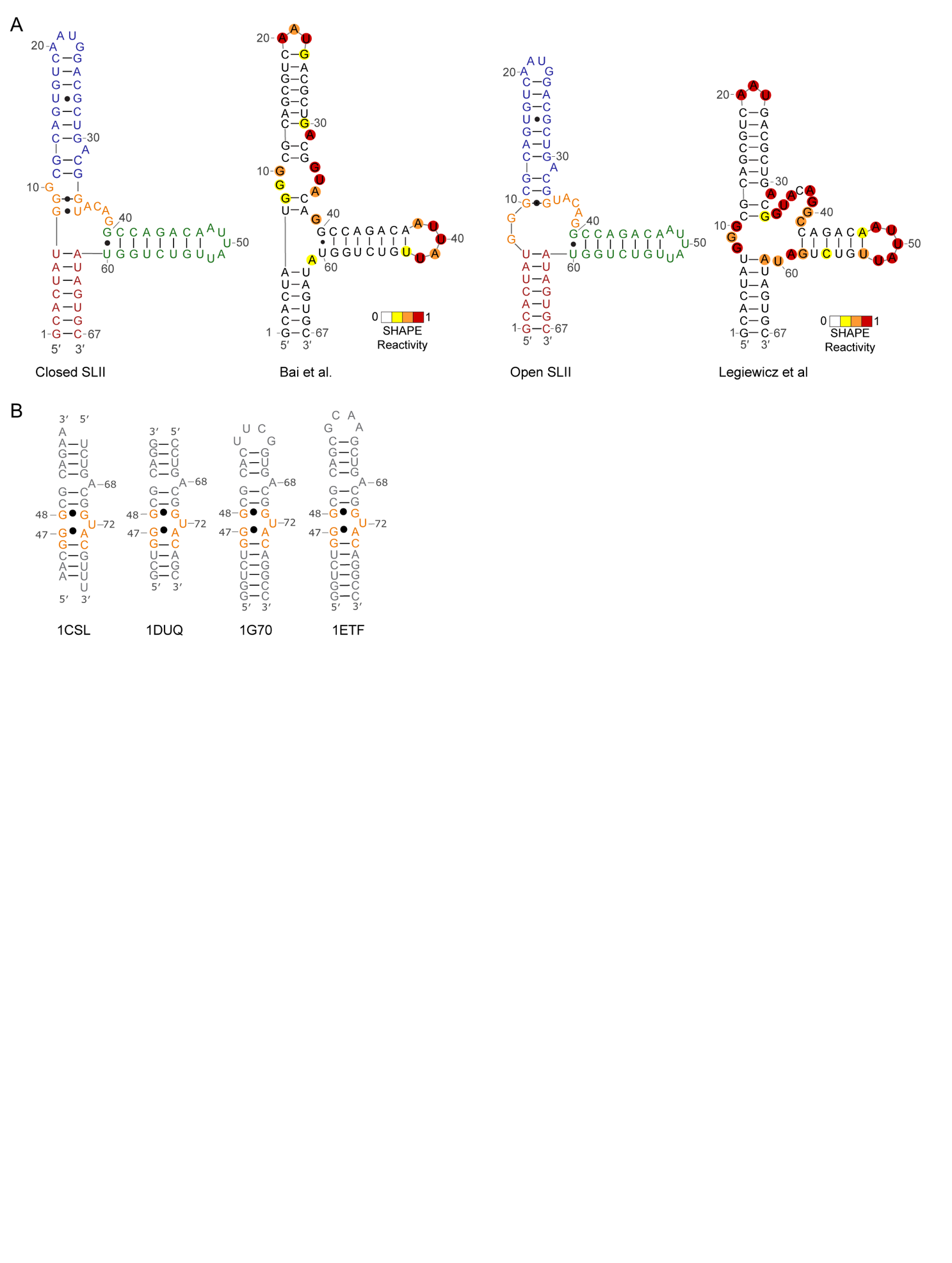
Fig S2. Secondary structures of RRE SLII. (A)** Comparison of the secondary structures of RRE SLII conformers with those predicted by SHAPE analysis. The nucleotides are numbered as in our SLII structure. Secondary structures of RRE predicted by SHAPE analyses differ at the 3-way junction in SLII (1)(2). In Bai et al., the GGG motif and antiparallel GUAC sequence forms an internal loop within stem IIB. This predicted secondary structure is akin to the closed SLII conformer. In Legiewicz et al., the GGG motif and GUACAG forms a 3-way junction as single stranded RNA, akin to the open SLII conformer. **(B)** Modified stem IIB structures used in NMR and crystallographic studies. The nucleotides in 3-way junction of RRE SLII are colored in orange. Nucleotides are numbered as reported in their respective publication (3-6). Since the high-affinity Rev binding sites is designed as an internal loop, the structures do not represent the 3-way junction of the native SLII structure.

**
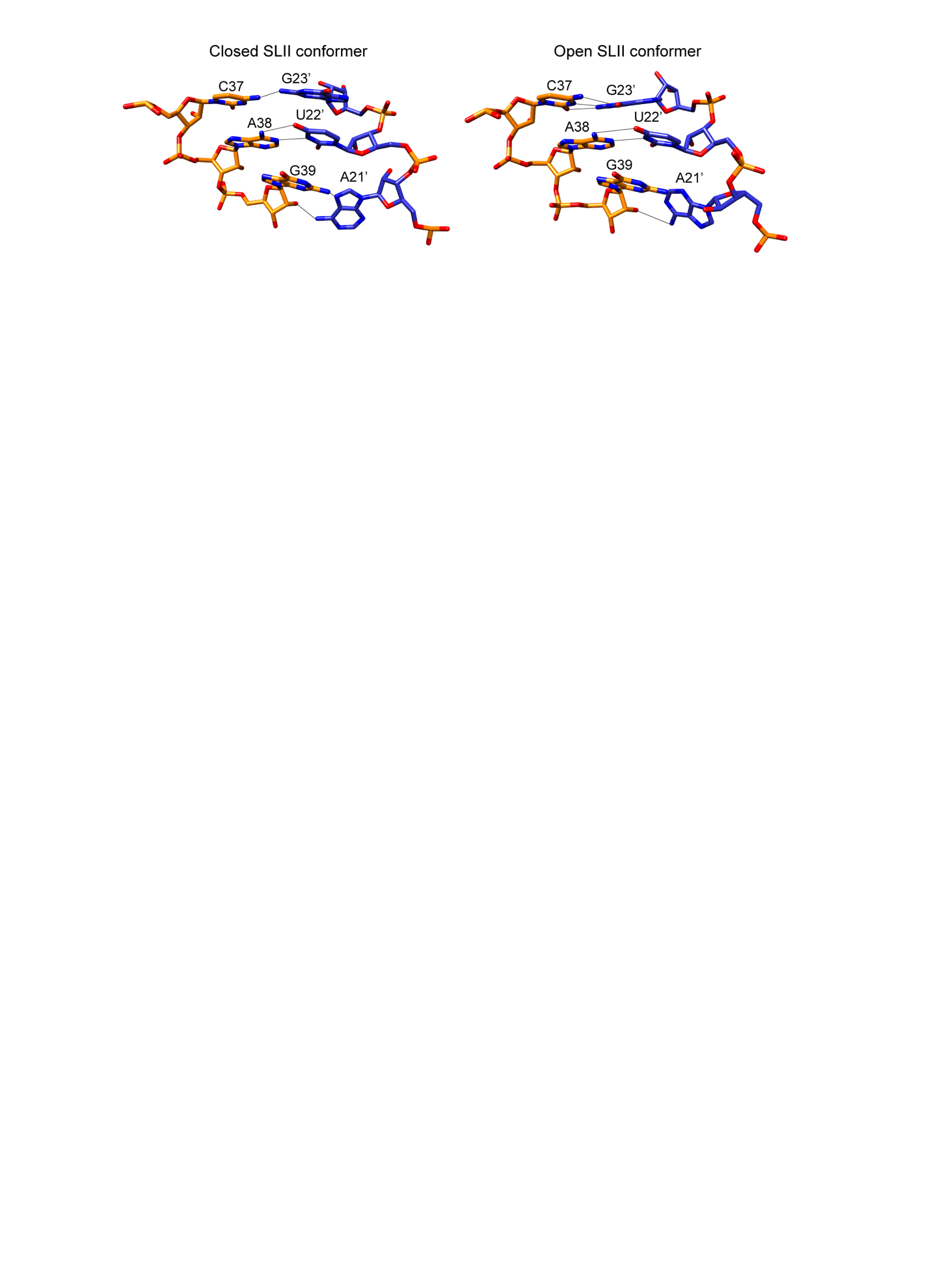
Fig S3. Crystal contacts between the 3-way junction and tetraloop of stem IIB**. The three-way junction nucleotides ^37^CAG^39^ (orange) form crystal contacts with the engineered ^20^AAUG^23^ apical loop of stem IIB of a symmetry related molecule (blue) in both the closed (left) and open (right) conformers. In both molecules, the ^37^CA^38^ base pairs with ^22^UG^23^ of the IIB tetraloop, and the sugar edge of G^39^ hydrogen bonds with the base of A^21^.

**
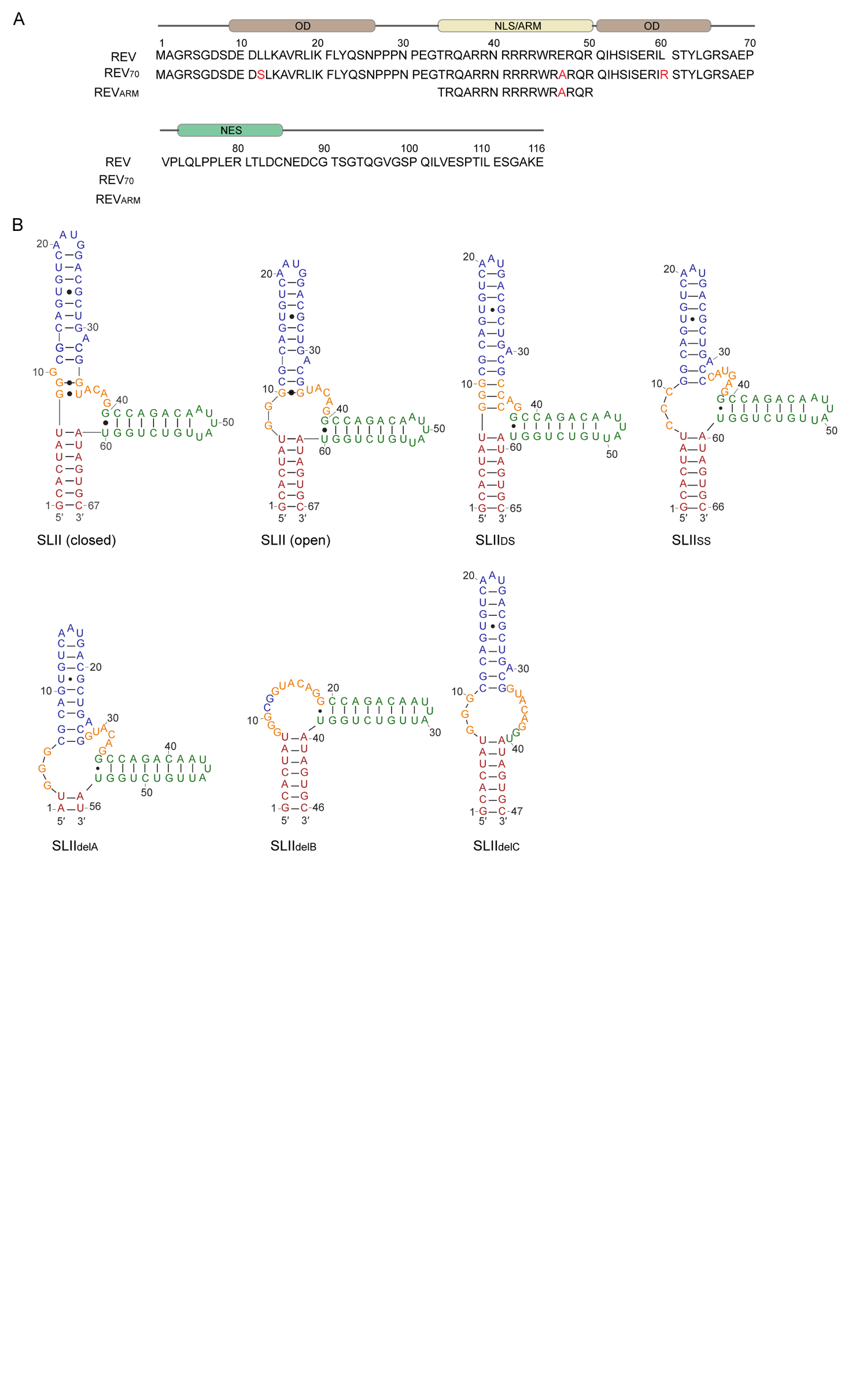
Fig S4. Rev protein and RRE SLII RNA sequences used in the binding assays.** **(A)** Rev proteins used in the EMSA binding assays. The top sequence is the Rev of HIV-1 group M subtype B (isolate HXB3, UniProt ID: P69718). Rev_70_ and Rev_ARM_ sequences are shown along with the mutations L12S, E47A, and L60R (red) (7,8). The protein domain arrangement is shown above the sequences. **(B)** SLII mutants used in EMSA. RNA sequences are colored as in the main text.

**References**

1. Bai, Y., Tambe, A., Zhou, K. and Doudna, J.A. (2014) RNA-guided assembly of Rev-RRE nuclear export complexes. *Elife*, **3**, e03656.

2. Legiewicz, M., Badorrek, C.S., Turner, K.B., Fabris, D., Hamm, T.E., Rekosh, D., Hammarskjöld, M.L. and Le Grice, S.F. (2008) Resistance to RevM10 inhibition reflects a conformational switch in the HIV-1 Rev response element. *Proc Natl Acad Sci U S A*, **105**, 14365-14370.

3. Battiste, J.L., Mao, H., Rao, N.S., Tan, R., Muhandiram, D.R., Kay, L.E., Frankel, A.D. and Williamson, J.R. (1996) Alpha helix-RNA major groove recognition in an HIV-1 rev peptide-RRE RNA complex. *Science*, **273**, 1547-1551.

4. Gosser, Y., Hermann, T., Majumdar, A., Hu, W., Frederick, R., Jiang, F., Xu, W. and Patel, D.J. (2001) Peptide-triggered conformational switch in HIV-1 RRE RNA complexes. *Nat Struct Biol*, **8**, 146-150.

5. Ippolito, J.A. and Steitz, T.A. (2000) The structure of the HIV-1 RRE high affinity rev binding site at 1.6 A resolution. *J Mol Biol*, **295**, 711-717.

6. Hung, L.W., Holbrook, E.L. and Holbrook, S.R. (2000) The crystal structure of the Rev binding element of HIV-1 reveals novel base pairing and conformational variability. *Proc Natl Acad Sci U S A*, **97**, 5107-5112.

7. Daugherty, M.D., Liu, B. and Frankel, A.D. (2010) Structural basis for cooperative RNA binding and export complex assembly by HIV Rev. *Nat Struct Mol Biol*, **17**, 1337-1342.

8. Jayaraman, B., Crosby, D.C., Homer, C., Ribeiro, I., Mavor, D. and Frankel, A.D. (2014) RNA-directed remodeling of the HIV-1 protein Rev orchestrates assembly of the Rev-Rev response element complex. *Elife*, **3**, e04120.
